# The Population Based Risk of Obstructive Sleep Apnea and Psychiatric Conditions

**DOI:** 10.1155/da/4329208

**Published:** 2025-11-11

**Authors:** Chuan-Yi Kao, Tsui-Hsein Huang, Chuan-Hui Kao, Jing-Yang Huang, Chia-Tze Kao, Yi Hsien Hsieh

**Affiliations:** ^1^Institute of Medicine, Chung Shan Medical University, Taichung, Taiwan; ^2^Department of Psychiatry, Chung Shan Medical University Hospital, Taichung, Taiwan; ^3^School of Dentistry, Chung Shan Medical University, Taichung, Taiwan; ^4^Department of Emergency Medicine, Chung Shan Medical University Hospital, Taichung, Taiwan; ^5^Department of Medical Research, Chung Shan Medical University Hospital, Taichung, Taiwan; ^6^Department of Dentistry, Chung Shan Medical University Hospital, Taichung, Taiwan; ^7^Dental Department, Chung Shan Medical University Hospital, Taichung, Taiwan

**Keywords:** anxiety, depression, obstructive sleep apnea, psychiatric disorders, retrospective cohort design, risk

## Abstract

**Background:**

This study investigates the association between obstructive sleep apnea (OSA) and the risk of developing psychiatric disorders. OSA, characterized by intermittent hypoxia and sleep fragmentation, contributes to brain damage and emotional regulation issues, which may predispose individuals to psychiatric conditions such as depression, anxiety, bipolar disorder, and schizophrenia. The research focuses on understanding the heightened risks of these disorders in OSA patients to inform clinical interventions. To assess the risk differences for psychiatric disorders in patients with OSA compared to those without OSA.

**Materials and Methods:**

The study utilized a retrospective cohort design, analyzing de-identified electronic health records (EHRs) from the TriNetX US Network. Data from 7,079,095 individuals (aged 18–65 years) were included. After exclusion (prior psychiatric disorders, circadian rhythm disorders, central sleep apnea, pregnancy, or death before index date), 368,125 OSA patients and 4,396,092 non-OSA individuals remained eligible. Following propensity score matching (PSM), 360,708 patients per group were analyzed. divided into OSA (368,125 patients) and non-OSA (4,396,092 patients) cohorts. PSM (360,708 patients per group) was applied to balance baseline characteristics. The primary outcome was the 8-year risk of newly diagnosed psychiatric disorders, analyzed using Cox proportional hazards models.

**Results:**

Patients with OSA showed significantly higher cumulative probabilities for psychiatric disorders over 8 years: depressive disorders: 27.4% in OSA patients vs. 15.8% in non-OSA patients (hazard ratio [HR]: 1.913). Anxiety disorders: 37.4% in OSA patients vs. 25.4% in non-OSA patients (HR: 1.663). Bipolar disorder: increased risk in OSA patients (HR: 1.885). Schizophrenia: minimal differences between groups (HR: 0.971). Subgroup analyses revealed that younger individuals and those with higher BMI were at greater risk for psychiatric disorders.

**Conclusion:**

OSA significantly elevates the risk of psychiatric disorders, particularly depression and anxiety. These findings emphasize the need for targeted screening and management strategies for high-risk populations, including younger and overweight individuals.

## 1. Introduction

The correct way to address a disease is by identifying its underlying cause. The causes of mental illnesses can be multifactorial. Excluding genetic and environmental factors, maintaining a healthy physiological state can help preserve mental well-being. Obstructive sleep apnea (OSA) is a widespread systemic disorder with far-reaching health implications, spanning cardiovascular, metabolic, and psychiatric domains. Research highlights that individuals with OSA experience increased risks for psychiatric conditions, including depression, anxiety, bipolar disorder, and schizophrenia [1–3].

OSA-induced sleep fragmentation disrupts the natural sleep architecture, especially impacting restorative deep sleep and REM sleep, both of which are vital for emotional regulation and memory processing. This disruption may underpin the observed link between OSA and mood disorders, as impaired REM sleep adversely affects emotional stability and the ability to manage negative emotions [4, 5]. Additionally, chronic sleep deprivation resulting from OSA is known to impair cognitive domains such as attention, executive function, and memory, which are central to mental health and are often compromised in conditions like depression, anxiety, and ADHD [6, 7].

From a psychiatric standpoint, emotional regulation dysfunction is pivotal in understanding the connection between OSA and psychiatric disorders. Chronic sleep loss, intermittent hypoxia, and associated neural damage diminish the capacity to cope with stress and manage daily challenges, often leading to irritability, heightened anxiety, and emotional instability [8]. Persistent fatigue may further promote feelings of helplessness and hopelessness, hallmarks of depressive states [9]. Epidemiological data indicate that individuals with OSA are two to three times more likely to experience depressive symptoms and are similarly predisposed to heightened anxiety due to concerns about sleep quality and daytime functioning [10, 11]. OSA's interference with daily activities can result in social withdrawal and reduced occupational performance, compounding mental health difficulties.

There is also an emerging interest in the link between OSA and bipolar disorder. Studies suggest that OSA prevalence is higher in individuals with bipolar disorder. Given the centrality of sleep regulation in bipolar disorder, disruptions caused by OSA can intensify emotional instability and increase the likelihood of mood episodes [12, 13]. Mental illnesses have complex origins, influenced by a variety of factors. More research is focusing on physiological and pathological contributors to these conditions. The prevalence of OSA is increasing in today's society, and it is recognized that OSA can lead to mental health issues due to alterations in the body's physiological structure. Some of these mental health disturbances can be treated and reversed, while others may not be. Understanding the underlying causes of mental illnesses is essential to delivering the most effective treatment.

This study was to evaluate the differences in psychiatric disorder risks between patients with OSA and those without. By analyzing the incidence of psychiatric disorders in these populations, we seek to inform clinical interventions designed to improve mental health outcomes in individuals affected by OSA.

## 2. Methods

### 2.1. Study Design

This study was a retrospective cohort analysis using global federated electronic health record (EHR) datasets to examine the risk of psychiatric disorders among adults diagnosed with OSA compared to adults without OSA.

### 2.2. Data Source

Data were extracted on May 21, 2024, from the TriNetX US Network, a global federated health research network that provides access to de-identified electronic medical records (EMRs) from healthcare organizations (HCOs). The dataset includes diagnostic, procedural, medication, laboratory, and genomic data, mainly sourced from large academic medical institutions across the United States. TriNetX is compliant with the Health Insurance Portability and Accountability Act (HIPAA) and is ISO 27001:2013 certified, ensuring data protection and regulatory compliance. TriNetX aggregates and de-identifies patient data, which enables secure web-based access to demographic, diagnostic, and procedural records. Data coding follows standardized biomedical ontologies such as SNOMED-CT and ICD-10, ensuring consistency in clinical terminology.

### 2.3. Study Population

**Figure 1:**
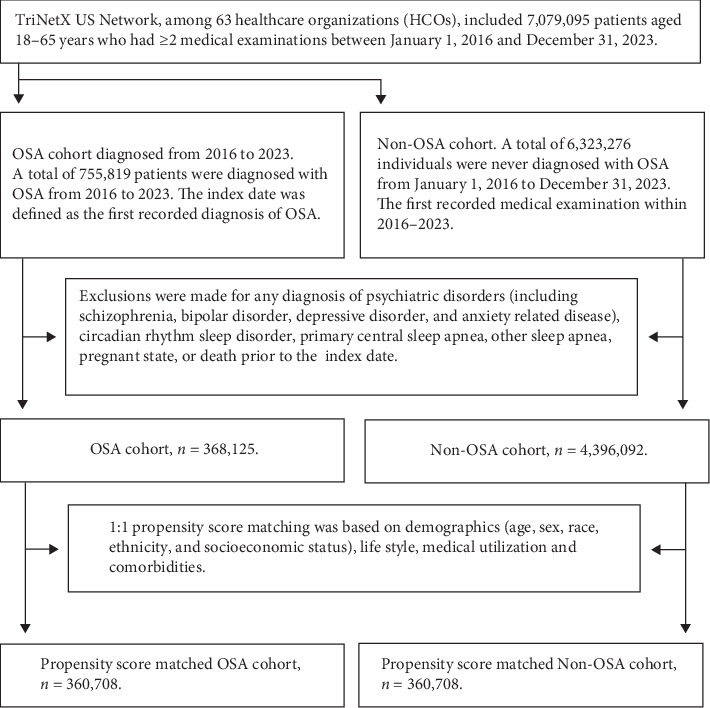
The sample selection flowchart and the propensity matching process.

We included adult patients aged 18–65 years with at least two medical examinations recorded between January 1, 2016 and December 31, 2023. A total of 7,079,095 individuals met these criteria. After exclusions, 368,125 OSA patients and 4,396,092 non-OSA patients remained, before matching.  Figure 1 illustrates the sample selection and refinement process, including the inclusion and exclusion criteria. This rigorous protocol minimized confounding factors and ensured a well-defined study population.

### 2.4. OSA Cohort

The cohort included 755,819 patients diagnosed with OSA (ICD-10: G47.33) within the specified timeframe. Exclusions applied to individuals with prior diagnoses of psychiatric disorders, circadian rhythm sleep disorders, other forms of sleep apnea, pregnancy, or records of death before the index date. After exclusions, 368,125 patients with OSA were retained for analysis.

### 2.5. Non-OSA Cohort

We identified 6,323,276 individuals without any record of OSA diagnoses during the study period. The index date was based on the first medical examination within this timeframe, and the same exclusion criteria were applied, resulting in a final sample of 4,396,092 individuals for the non-OSA group.

### 2.6. Study Outcomes and Follow-Up

The primary outcome was the 8-year risk of newly diagnosed psychiatric disorders, including schizophrenia, bipolar disorder, depressive disorder, and anxiety-related conditions. Outcomes were tracked from the index date, and data were analyzed using methods consistent with observational cohort study designs.

### 2.7. Ethics and Approval

All data were de-identified per HIPAA Privacy Rule standards (§164.514(a)), with de-identification verified by a qualified expert as outlined in §164.514(b)(1). Due to the use of de-identified data, Institutional Review Board (IRB) approval was waived. Additionally, approval for the study was obtained from the IRB of Chung Shan Medical University Hospital (CSMUH), under the approval number CS2-21176.

### 2.8. Statistical Analysis

All statistical analyses were conducted using TriNetX Analytics platform, which supports robust data analysis and visualization functions suitable for observational studies. We applied propensity score matching (PSM) to balance baseline characteristics between OSA and non-OSA cohorts to reduce potential confounding. Propensity scores were calculated using logistic regression, taking into account demographic variables, comorbidities, and relevant medical history.

After matching, we estimated hazard ratios (HRs) with 95% confidence intervals (CIs) for the primary outcomes—psychiatric disorders—including schizophrenia, bipolar disorder, depressive disorder, and anxiety-related conditions. Cox proportional hazards models were utilized to analyze time-to-event data for each psychiatric outcome over the 8-year follow-up period.

We evaluated the assumption of proportional hazards for each outcome, and any violations were addressed through stratification or time-varying covariates. Statistical significance was defined as a two-tailed *p*-value of less than 0.05. Sensitivity analyses were performed to assess the robustness of our results, including adjustments for additional covariates and subgroup analyses based on age, sex, and comorbidity burden. All analyses were completed on de-identified data sets, ensuring compliance with HIPAA regulations and maintaining patient confidentiality.

## 3. Results

### 3.1. Characteristics of the Study Cohorts

After propensity-score matching, 360,708 patients in each cohort (OSA and non-OSA) were selected. Significant baseline differences before matching were effectively minimized, producing balanced groups for analysis.

### 3.2. Baseline Characteristics Before Matching

The baseline characteristics of patients with OSA and non-OSA patients before PSM, shown in Table 1. Significant differences are evident in age, gender, race, and health conditions, with standardized mean differences (SMDs) indicating the extent of variation. These discrepancies highlight the need for matching to control for confounding factors.

### 3.3. Baseline Characteristics After Matching

Post-PSM shows the adjusted baseline characteristics, where SMD values are notably reduced, demonstrating that the groups are now balanced in terms of demographics and health factors. This balancing ensures that any differences in psychiatric disorder risk are more attributable to OSA rather than baseline disparities.

### 3.4. Cumulative Probability and HRs

The cumulative probability and HRs for the occurrence of psychiatric disorders in OSA and non-OSA patients over 1, 3, and 8 years were summarized in Table 2. The higher HRs in the OSA group suggest an increased risk for psychiatric conditions, supporting a potential association between OSA and mental health risks.

### 3.5. Risk of Psychiatric Disorders Between OSA and Non-OSA Patients

**Figure 2:**
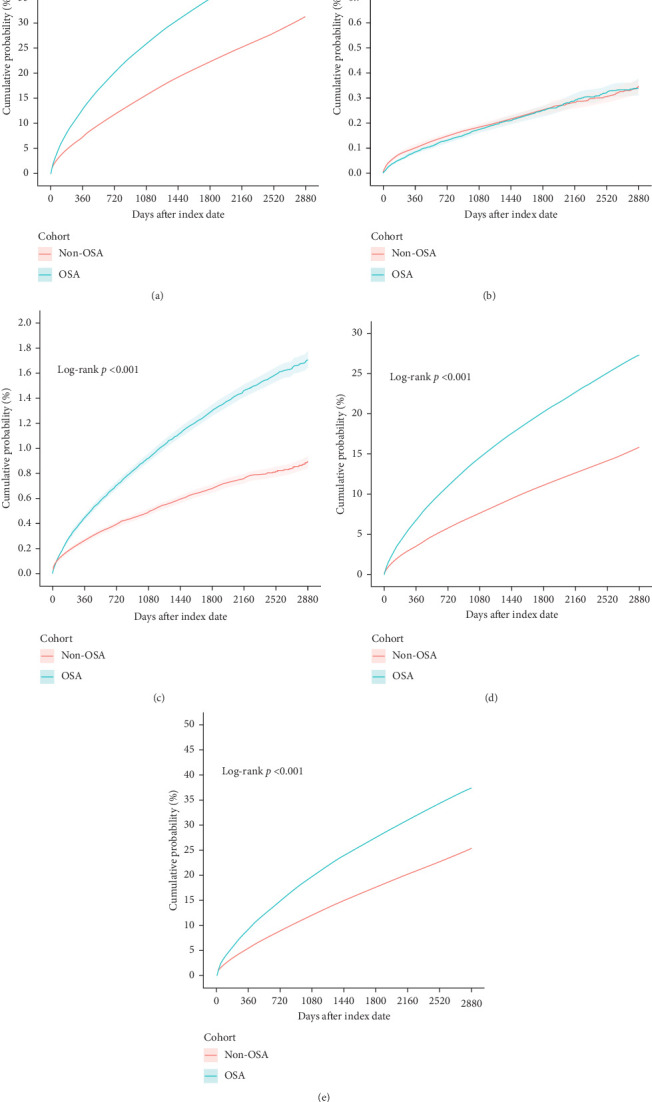
The risk of psychiatric disorders between OSA and non-OSA patients. (A) Composite psychiatric disorders, (B) schizophrenia, (C) bipolar disorder, (D) depressive disorders, and (E) anxiety-related diseases.

The comparative data on the risk of various psychiatric disorders, such as depression and anxiety, between OSA and non-OSA patients shown in Figure 2. Figure 2A–E shows a significantly higher incidence of psychiatric disorders in OSA patients, especially on depressive disorders and anxiety-related diseases.

### 3.6. Subgroup Analysis by Demographic Factors

**Figure 3:**
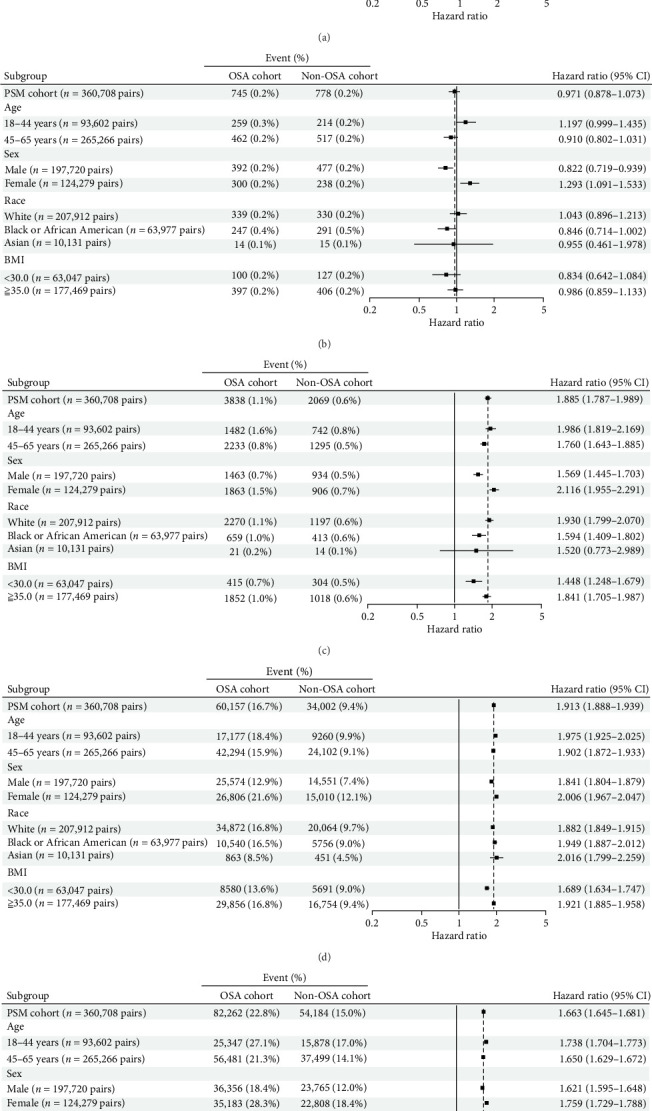
Subgroup analysis for risk of (A) composite psychiatric disorders, (B) schizophrenia, (C) bipolar disorder, (D) depressive disorders, and (E) anxiety-related diseases in patients with OSA stratified by age, sex, race, and BMI.

The subgroup analysis of psychiatric disorder risks by age, sex, race, and BMI shown in Figure 3. This stratified analysis reveals that certain subgroups, such as younger patients or those with higher BMIs, have elevated risks, aiding in identifying high-risk populations for targeted interventions.

### 3.7. Composite Psychiatric Disorders

OSA patients showed a 1-year cumulative probability of 12.7%, rising to 45.0% at 8 years. In comparison, the non-OSA cohort had a cumulative probability of 7.2% at 1 year and 31.3% at 8 years (HR: 1.694; Figures 2A and 3A). When comparing the factors of age, sex, race, and BMI, patients with OSA show a higher risk value than non-OSA patients. All CI that does not cross 1 suggests statistical significance.

### 3.8. Schizophrenia

In the schizophrenia HR statistics, when comparing the male sex factor, OSA patients show a higher risk value than non-OSA patients. Minimal difference was observed between the cohorts, with an HR of 0.971 (Figures 2B and 3B).

### 3.9. Bipolar Disorder

In the statistics for bipolar disorder, all factors show differences except for the Asian race factor, which shows no difference. In comparison across these factors, patients with OSA have a higher risk than non-OSA patients. The cumulative incidence in OSA patients reached 1.7% at 8 years (HR: 1.885; Figures 2C and 3C).

### 3.10. Depressive Disorders

In the HR statistics for depressive disorders, comparisons across factors such as age, sex, race, and BMI show that patients with OSA have a higher risk than non-OSA patients. At 8 years, cumulative incidence was 27.4% in OSA patients versus 15.8% in non-OSA patients (HR: 1.913; Figures 2D and 3D).

### 3.11. Anxiety Disorders

In the HR statistics for anxiety-related diseases, comparisons across factors such as age, sex, race, and BMI show that patients with OSA have a higher risk than non-OSA patients. Cumulative incidence for OSA patients was 37.4% at 8 years compared to 25.4% in non-OSA patients (HR: 1.663; Figures 2E and 3E).

## 4. Discussion

The results of this study highlight the substantial psychiatric risks associated with OSA, especially regarding depressive and anxiety disorders. Elevated HRs for depressive disorders (1.913) and anxiety disorders (1.663) are consistent with existing research linking sleep disturbances and intermittent hypoxia to emotional dysregulation and cognitive issues. Intermittent hypoxia was found to increase anxiety and depressive-like behaviors in a pulmonary fibrosis model, indicating that oxygen deprivation could intensify emotional dysregulation [14].

The mechanisms through which OSA contributes to psychiatric disorders are likely multifaceted, involving neuroinflammatory responses and oxidative stress induced by intermittent hypoxia. This hypoxia, combined with sleep fragmentation, disrupts the normal sleep architecture, impairing cognitive processes and heightening vulnerability to mood disorders. Our result was similar with one study showed that approximately 65% of OSA patients have a psychiatric comorbidity, with depressive disorders being the most common (31.5%) [15].

Subgroup analyses revealed that younger OSA patients and those with higher BMIs had an elevated risk of depressive and anxiety disorders, suggesting demographic-specific vulnerabilities. These findings may inform targeted psychiatric screenings in high-risk subpopulations, improving early detection and management. Our findings also support integrative care models, where sleep specialists collaborate with psychiatrists. Prior research shows that continuous positive airway pressure (CPAP) therapy reduces depressive and anxiety symptoms in OSA patients [16]. The present study results indicated that while OSA patients had a slightly higher risk of schizophrenia compared to non-OSA patients, the difference between the two groups was minimal. Specifically, the HR for OSA patients relative to non-OSA patients was 0.971, indicating a negligible increase in risk. This finding aligns with other study showing that while OSA is associated with various psychiatric disorders, the additional risk specifically for schizophrenia in male patients with OSA is not substantially elevated [17]. This minor increase in HR suggests that, although there is a risk association, it may not be clinically significant in this cohort.

OSA prevalence is elevated among individuals with bipolar disorder, ranging from 15% to 30%, likely due to sleep regulation disturbances intrinsic to bipolar disorder [18]. The statistical analyses revealed that differences exist across multiple factors, with the exception of the Asian race factor, which showed no significant difference in bipolar disorder risk. When comparing the risk between patients with OSA and those without, the study found that OSA patients had a higher risk of developing bipolar disorder over time. The cumulative incidence for bipolar disorder in OSA patients reached 1.7% after 8 years, with a HR of 1.885, indicating a substantially increased risk relative to non-OSA patients. This aligns with findings that suggest a strong association between OSA and increased vulnerability to psychiatric disorders, including bipolar disorder, particularly over extended observation periods [19]. This study confirms that patients with OSA have a significantly elevated risk of developing depression compared to individuals without OSA, even after adjusting for factors like age, sex, race, and BMI. Over an 8-year period, the cumulative incidence of depressive disorders in OSA patients reached 27.4%, contrasted with 15.8% in non-OSA individuals, with a HR of 1.913, indicating a markedly increased likelihood of depression in the OSA group. One analysis reported an even higher HR of 2.18 (95% CI = 1.55–3.08), further confirming the significant association between OSA and depressive disorders [20]. Additional studies have similarly highlighted a correlation between sleep apnea and heightened vulnerability to depressive symptoms [21–23].

Furthermore, OSA patients also show a significantly higher risk of anxiety disorders than non-OSA individuals. Specifically, the HR for anxiety disorders among OSA patients was 1.663, with a cumulative incidence of 37.4% over 8 years, compared to 25.4% for those without OSA. This finding underscores the greater susceptibility of OSA patients to anxiety-related conditions, which may also be influenced by age, sex, and BMI. Evidence from other research supports that CPAP therapy can alleviate symptoms of both depression and anxiety in OSA patients, suggesting that proper management of sleep apnea may lead to improved psychiatric outcomes [24].

Despite the advantages of a large dataset and matching methods, the current study has limitations. Intellectual disability was not identifiable in the dataset and could not be excluded, which may represent a potential confounder [25]. Notably, it relies on diagnostic codes, which can result in the underreporting or misclassification of psychiatric disorders, potentially impacting data accuracy. Future research should adopt a longitudinal approach to investigate the mechanistic pathways that link OSA with mental health disorders, particularly focusing on neurocognitive and inflammatory biomarkers. Although many studies have identified associations between OSA and various mental illnesses, persistent findings suggest a substantial relationship likely influenced by factors such as the ambiguous etiology of psychiatric conditions, suboptimal treatments, and the rising prevalence of OSA. Additionally, this study has not explored changes in mental health outcomes in OSA patients following treatment interventions, such as CPAP, oral appliances, or surgical methods, due to incomplete data on treatment efficacy. Future studies should address these gaps to clarify the effects of therapeutic strategies on mental health in OSA patients.

## 5. Conclusion

This study underscores the significant psychiatric risks associated with OSA, revealing an increased likelihood of developing mental health disorders such as depression, anxiety, bipolar disorder, and schizophrenia. Analysis of an extensive patient dataset using PSM indicates that OSA patients have a higher cumulative probability of psychiatric disorders over time, especially in younger individuals or those with higher BMIs. It is suggested that addressing OSA may be crucial for preventing or mitigating psychiatric conditions.

## Figures and Tables

**Table 1:** The comparison of baseline characteristics before and after matching.

Item	Before PSM			After PSM		
	OSA	Non-OSA	SMD	OSA	Non-OSA	SMD
*N*	366203	4370072	—	360708	360708	—
Age at index date	50.4 ± 10.7	42.4 ± 14.2	0.6341	50.3 ± 10.7	50.6 ± 10.8	0.0286
Sex						
Female	129109 (35.3%)	2533392 (58.0%)	0.4676	128281 (35.6%)	126160 (35.0%)	0.0123
Male	208569 (57.0%)	1574211 (36.0%)	0.4292	204680 (56.7%)	207306 (57.5%)	0.0147
Ethnicity						
Not Hispanic or Latino	263416 (71.9%)	2937629 (67.2%)	0.1025	259257 (71.9%)	259248 (71.9%)	0.0001
Hispanic or Latino	23968 (6.5%)	435004 (10.0%)	0.1242	23814 (6.6%)	23496 (6.5%)	0.0036
Race						
White	220872 (60.3%)	2660481 (60.9%)	0.0116	217627 (60.3%)	219139 (60.8%)	0.0086
Black or African American	65819 (18.0%)	634374 (14.5%)	0.0938	64924 (18.0%)	63052 (17.5%)	0.0136
Asian	10746 (2.9%)	231905 (5.3%)	0.1196	10651 (3.0%)	11963 (3.3%)	0.0209
Socioeconomic and psychosocial circumstances	3691 (1.0%)	27797 (0.6%)	0.0412	3522 (1.0%)	3185 (0.9%)	0.0097
BMI						
<30	63571 (17.4%)	1518978 (34.8%)	0.4044	63312 (17.6%)	65842 (18.3%)	0.0183
≧30	182281 (49.8%)	804860 (18.4%)	0.7010	177307 (49.2%)	176306 (48.9%)	0.0056
Lifestyle						
Nicotine dependence	26213 (7.2%)	150159 (3.4%)	0.1668	25171 (7.0%)	24965 (6.9%)	0.0022
Alcohol related disorders	5804 (1.6%)	37496 (0.9%)	0.0662	5592 (1.6%)	5160 (1.4%)	0.0099
Baseline medical utilization						
Outpatient services	231755 (63.3%)	1485097 (34.0%)	0.6132	226724 (62.9%)	230373 (63.9%)	0.0210
Preventive medicine Services	64217 (17.5%)	1481980 (33.9%)	0.3814	64063 (17.8%)	65238 (18.1%)	0.0085
Emergency department Services	44913 (12.3%)	350123 (8.0%)	0.1412	43312 (12.0%)	42850 (11.9%)	0.0039
Inpatient Encounter	20369 (5.6%)	84331 (1.9%)	0.1922	19053 (5.3%)	18073 (5.0%)	0.0123
Baseline comorbidity						
Hypertensive diseases	179369 (49.0%)	609018 (13.9%)	0.8150	174080 (48.3%)	175119 (48.5%)	0.0058
Disorders of lipoprotein metabolism	135310 (36.9%)	512858 (11.7%)	0.6146	130891 (36.3%)	131712 (36.5%)	0.0047
Diabetes mellitus	80520 (22.0%)	246570 (5.6%)	0.4876	76942 (21.3%)	75604 (21.0%)	0.0091
Chronic lower respiratory diseases	55337 (15.1%)	193621 (4.4%)	0.3657	51514 (14.3%)	49610 (13.8%)	0.0152
Ischemic heart diseases	35390 (9.7%)	82160 (1.9%)	0.3385	33009 (9.2%)	32053 (8.9%)	0.0093
Acute kidney failure and chronic kidney disease	29048 (7.9%)	101207 (2.3%)	0.2568	27519 (7.6%)	25516 (7.1%)	0.0213
Diseases of arteries, arterioles and capillaries	16801 (4.6%)	62415 (1.4%)	0.1858	15903 (4.4%)	15139 (4.2%)	0.0104
Fatty liver	12038 (3.3%)	30907 (0.7%)	0.1852	11719 (3.2%)	6443 (1.8%)	0.0935
Cerebrovascular diseases	11422 (3.1%)	41905 (1.0%)	0.1533	10879 (3.0%)	10850 (3.0%)	0.0005
Fibrosis and cirrhosis of liver	3572 (1.0%)	20521 (0.5%)	0.0598	3425 (1.0%)	4014 (1.1%)	0.0162
Cannabis related disorders	1586 (0.4%)	13217 (0.3%)	0.0216	1520 (0.4%)	1701 (0.5%)	0.0075
Opioid related disorders	1239 (0.3%)	9634 (0.2%)	0.0223	1183 (0.3%)	1321 (0.4%)	0.0065
Alcoholic liver disease	1141 (0.3%)	11610 (0.3%)	0.0086	1095 (0.3%)	1875 (0.5%)	0.0338
Sedative, hypnotic, or anxiolytic related disorders	84 (0.0%)	702 (0.0%)	0.0049	82 (0.0%)	85 (0.0%)	0.0005

*Note:* Baseline period was defined as the time interval within 6 months before index date. Propensity score matching was based on demographics (age, sex, race, ethnicity, and socioeconomic status), life style, medical utilization, and comorbidities.

**Table 2:** The cumulative probability and hazard ratio for risk of psychiatric disorders after index date among propensity score matched OSA cohort and non-OSA cohort.

Item	Event	Cumulative probability (95% CI) of psychiatric disorders			Hazard ratio (95% CI)
		1-year	3-year	8-year	
Composite psychiatric disorders					
OSA cohort (*n* = 360,708)	103,960	12.7% (12.8%–12.6%)	25.9% (26.1%–25.8%)	45.0% (45.3%–44.7%)	1.694 (1.678–1.710)
Non-OSA cohort (*n* = 360,708)	68,885	7.2% (7.3%–7.1%)	15.7% (15.8%–15.5%)	31.3% (31.5%–31.0%)	Reference
Schizophrenia					
OSA cohort (*n* = 360,708)	745	0.1% (0.1%–0.1%)	0.2% (0.2%–0.2%)	0.3% (0.4%–0.3%)	0.971 (0.878–1.073)
Non-OSA cohort (*n* = 360,708)	778	0.1% (0.1%–0.1%)	0.2% (0.2%–0.2%)	0.3% (0.4%–0.3%)	Reference
Bipolar disorder					
OSA cohort (*n* = 360,708)	3,838	0.4% (0.5%–0.4%)	0.9% (1.0%–0.9%)	1.7% (1.8%–1.6%)	1.885 (1.787–1.989)
Non-OSA cohort (*n* = 360,708)	2,069	0.3% (0.3%–0.2%)	0.5% (0.5%–0.5%)	0.9% (0.9%–0.8%)	Reference
Depressive disorders					
OSA cohort (*n* = 360,708)	60,157	6.7% (6.8%–6.7%)	14.6% (14.7%–14.4%)	27.4% (27.7%–27.1%)	1.913 (1.888–1.939)
Non-OSA cohort (*n* = 360,708)	34,002	3.5% (3.5%–3.4%)	7.7% (7.8%–7.6%)	15.8% (16.0%–15.6%)	Reference
Anxiety-related diseases					
OSA cohort (*n* = 360,708)	82,262	9.1% (9.2%–9.0%)	19.7% (19.9%–19.6%)	37.4% (37.6%–37.1%)	1.663 (1.645–1.681)
Non-OSA cohort (*n* = 360,708)	54,184	5.3% (5.4%–5.2%)	12.0% (12.1%–11.9%)	25.4% (25.7%–25.2%)	Reference

## Data Availability

The data used in this study were extracted on May 21, 2024, from the TriNetX US Network, a global federated health research platform that provides access to de-identified electronic medical records (EMRs) from participating healthcare organizations. Due to data use agreements and privacy regulations, these data are not publicly available but can be accessed through TriNetX upon reasonable request and subject to approval.
